# Control of oncogenesis and cancer therapy resistance

**DOI:** 10.1038/sj.bjc.6601552

**Published:** 2004-02-03

**Authors:** R Perona, I Sánchez-Pérez

**Affiliations:** 1 Instituto de Investigaciones Biomédicas CSIC-UAM, C/Arturo Duperier, 4, Madrid 28029, Spain

**Keywords:** oncogenes, drug resistance, radiotherapy, apoptosis, chemotherapy

## Abstract

Despite the combined action of surgery, radiotherapy and chemotherapy, the leading cause of death in cancer patients continues to be the acquired, or intrinsic, tumour resistance to therapy. Some of the genetic alterations that contribute to the malignant transformation are involved in maintaining cell survival under uncontrolled growth conditions. Chemotherapy agents, as well as radiotherapy, trigger a series of signalling pathways in the cells that activate not only the apoptotic machinery, but also cell-survival pathways. In this scenario, the efficacy of therapy is the result of balance between the apoptotic and the survival pathways activated in the tumour, and those elicited by the therapeutic agent. Apoptosis is one of the programmes usually altered in most cancers so as to guarantee tumour progression and, often, these alterations are responsible for therapy resistance, as well.

## ONCOGENIC TRANSFORMATION AND SURVIVAL PATHWAYS

Therapy resistance mechanisms that tumour cells develop are complex and can be acquired during, or after, treatment. This aggressive behaviour is often derived from survival pathways activated during malignancy progression and driven by oncogenic transformation. There are several examples of oncogenes that can activate such survival pathways, and among these are ras, src, raf, kit, together with the amplification of HER2 and EGFr. The paradigm of these oncogenes is ras, because of the involvement of mutations in ras itself in about 20% of human tumours. In other cases, EGFr overexpression or HER2-neu amplification positively regulate ras signalling pathways (Downward, 2003). Both, EGFr and Her2 expression are altered in many types of cancer, including those of the breast, ovary and stomach. Additionally, truncation of the EGFr, which results in deletion of the extracellular domain, is found in a significant proportion of glioblastomas and other tumour types. The EGFr family of tyrosine kinases is also activated by the autocrine production of EGF-like factors, such as TGF-*α*, in tumours. In addition to the raf/MAPK pathway involved in cell cycle activation, ras directly activates the catalytic subunit of the type I phosphatidylinositol-3 kinase (PI3K) that leads to the production of phosphatidylinositol 3,4,5 triphosphate, a second messenger that interacts with a large number of downstream enzymes such as AKT/PKB. AKT has a strong antiapoptotic action by phosphorylating several targets, and is the main survival pathway activated by ras. The mechanism by which AKT protects cells from apoptosis is by its ability to phosphorylate several components of the cell-death machinery (Vivanco and Sawyers, 2002). AKT inhibits the catalytic activity of caspases by phosphorylation. Bad, a proapoptotic member of the bcl2 family of proteins, is also phosphorylated by AKT, thus preventing its interaction with bclxl and abolishing its proapoptotic function. AKT also phosphorylates FKHR, a member of the forkhead family of transcription factors, thus preventing its nuclear translocation and inhibiting expression of its targets that include several proapoptotic proteins such as FasL and Bim. Finally, AKT phosphorylates p65, a subunit of the NFKB transcription factor, thus enhancing its transactivation potential and facilitating the expression of several antiapoptotic genes such as BCLxl, and the cIAP family of caspase inhibitors.

Different components of the PI3K pathway are also mutated in several types of tumours. The gene encoding p105, the catalytic subunit of PI3K, is found to be amplified in some ovarian cancers, while AKT has been observed to be amplified in ovarian and breast cancer (Ruggeri *et al*, 1998). PTEN, a 3-lipid phosphatase that converts PIP3 back to PIP2, acting as a suppressor gene, is deleted or mutated in several human cancers including those of the breast and endometrium, hepatocellular carcinomas, glioblastomas, lung cancer, renal carcinoma and thyroid tumours (Ali *et al*, 1999).

Several preclinical studies have reported a direct correlation between oncogenic transformation and drug resistance. Examples are the increase in resistance to radiation in bladder carcinomas induced by ras mutations, to CPT-11 and gemcitabine in colon cancer, to gemcitabine in pancreatic carcinoma, and to radiation and BCNU in glioblastomas (Chakravarti *et al*, 2002). In most of these cancers, resistance to therapy is due to upregulation of PI3K activity. In breast cancer, amplification of HER2 is associated with taxol resistance (Witters *et al*, 2003), and in glioblastomas increase in EGFr activity to radiation and BCNU (Chakravarti *et al*, 2002).

Some clinical studies have shown correlations between ras and myc mutations and resistance to therapy in ovarian cancer, and overexpression of EGFr in breast cancer. However, further studies are needed to provide a better overview of the problem, at the clinical level.

c-kit is a type II tyrosine kinase that activates different signalling pathways in response to the ligand: stem cell factor. Mutations in the catalytic activity domain in the cytoplasmic region are associated with transformation in patients with gastrointestinal tumours (GIST), a very resistant type of cancer (Hirota *et al*, 1998). Among the different signalling pathways associated with c-kit activation is the PI3K/Akt survival pathway that contributes to resistance to the different treatments.

p53, a tumour-suppressor gene, plays a central role in DNA damage detection, cell cycle transitions and apoptosis control in response to damage. Since most of the treatment protocols include DNA-damaging agents, the function of p53 is very important in therapeutic response. About 50% of all human cancers harbour mutations in the p53 gene, which affect its ability to activate expression of proapoptotic genes. Conversely, tumours that retain wild-type p53 frequently have modifications and/or defects in the pathways that facilitate the stabilisation of p53 in response to stress. p53 regulates transcription of genes that regulate both the mitochondrial as well as the receptor-activated apoptotic pathways such as APAf-1, Bax, NOXA, PERP, PTEN, PUMA, FAS, etc (Schuler and Green, 2001). p53-mediated activation of apoptotic target genes is also regulated by cofactors such as JMY, ASPP, and other members of the p53 family, p63 and p73. Both the JMY as well as the ASPP family of proteins have been shown, recently, to interact with p53 and to enhance the promoters of apoptotic target genes such as bax. IASPP1 is an evolutionary-conserved inhibitor of p53 that works as a transforming protein which co-operates with ras, E1A and E7 to transform cells *in vitro*. Enforced expression of IASPP confers resistance to radiation and cisplatin-induced apoptosis. IASPP expression is upregulated in human breast carcinomas expressing wt p53 and normal levels of ASPP (Bergamaschi *et al*, 2003).

At the clinical level, the role of p53 and drug resistance has been extensively evaluated by assessing clinical status in relation to response to chemotherapy and overall survival. Although in some studies correlations have been found between tp53 mutations and chemoresistance, such as in lung and ovarian carcinomas, in other types of tumours, the correlation has not been as clear and is due, possibly, to the involvement of other genetic changes that have accumulated in these tumours.

## SIGNAL TRANSDUCTION PATHWAYS ACTIVATED BY THERAPY: APOPTOSIS *VS* SURVIVAL

Apart from surgery, cancer treatment is based, mainly, on chemotherapy and radiotherapy, and both are cytotoxic agents that induce apoptosis in target cells. Since most cancers are not sensitive to these treatments, the resistance that is developed becomes a major problem at the clinical level. Further, when tumours relapse, the response to treatment is lower than that of the initial tumour. Most antitumour drugs, and also *γ*-radiation, are DNA-damaging agents. Since cells are routinely exposed on a daily basis to many such damaging agents, the principal targets of which are proteins and DNA, they develop different mechanisms in an attempt to minimise, or to resolve, any damage before the mitotic process commences. This is so as to pre-empt deleterious mutations being inherited in the daughter cells. Exposure of cells to therapeutic genotoxic agents initiates a series of events leading to activation of a wide range of signalling pathways that regulate cell cycle repair as well as apoptosis, and involves a wide group of genes with diverse functions. Current knowledge of cancer indicates that it is a complicated disease in which, depending on the type of tumour, many pathways are deregulated. Chemotherapy and radiation activate signalling pathways which can be rapidly induced, as well, in response to mitogenic stimulation and which, sometimes, are the same as those that are deregulated in the tumour. Consequently, it is important for the clinician to know the mechanisms by which apoptosis can be induced so that the balance of the signalling pathways may be tipped towards apoptosis.

Although the primary intracellular targets –of action of chemotherapy agents are different, it has become evident that induced cytotoxicity converges, ultimately, on a common pathway that induces apoptosis. Most of these pathways involve the activation of protein kinases. These include the nonreceptor tyrosine kinase c-abl, Lyn, the mitogen-activated protein kinase (MAPK) family, the transcription factor NFkB and the checkpoint proteins. In evolutionary terms, the MAPK pathway is highly conserved, which includes the ERK, JNK and p38 family. These are organised in a module of three kinases that are activated by a phosphorylation cascade. Each MAP kinase subfamily is activated by a specific upstream MAP kinase kinase (MKKs), which phosphorylates the threonine as well as the tyrosine residues within a conserved T–X–Y motif. Activation of the MAP kinase family members leads to phosphorylation of several cellular effector molecules, including protein kinases such as MAPKAPK1, MAPKAP-K2/3, Mnk1/2, as well as transcription factors such as c-jun, ATF-2, MEF2c and CHOP, all of which influence the fate of the cell.

The MAPKs are conserved proteins that regulate the growth, division and death of the cell. The MAPK pathway represents a cascade of phosphorylation events including three pivotal kinases, namely Raf, MEK (MAP kinase kinase) and ERK (MAP kinase). Activation of ERK by chemotherapy agents is controversial and, ultimately, depends on the type of tumour. Cisplatin, an antitumour agent that is widely used in the treatment of several kinds of solid tumours has been shown to cause activation of ERK in ovarian carcinoma (Hayakawa *et al*, 1999), neuroblastoma and HeLa cells, but not in keratinocytes. Inhibition or ERK activation results in increased sensitivity to cisplatin and taxol-induced cell death in ovarian cancer cell lines and some melanoma cell lines (Mandic *et al*, 2001), but can result in an increased resistance to cisplatin in HeLa cells.

Activation of the Jun-N-terminal kinase (JNK) pathway has been implicated in apoptotic response to DNA damage, cell stress and cytotoxic drugs. It appears that, in normal cells, the activation of JNK is a proapoptotic event and drives the activation of proapoptotic members of Bcl-2 family, causing an induction of cytochrome *c* release from the mitochondria. However, there is less consensus regarding the roles of JNK and of c-Jun in tumour cells. The initiation of mitochondrial apoptosis pathways by JNK is independent of its transcription effects, for the most part. In certain cell types, c-Jun overexpression is clearly a basis for resistance to DNA-damaging drugs, and resistance reversal has been observed using antisense RNA against c-Jun (Hayakawa *et al*, 1999). Conversely, in other cell systems, the absence of c-Jun makes cells more resistant to cisplatin treatment (Sanchez-Pérez and Perona, 1999). As such, there is still considerable controversy regarding JNK activation and its role in apoptosis and/or survival. Both mechanisms are not mutually exclusive, since certain parameters, such as the timing of the signal, could be important. Sustained JNK activation is required for apoptotic signalling and is sufficient for apoptosis in response to cisplatin and irradiation. In contrast, TNF-*α* causes transient JNK activation and apoptosis is not induced in some cell lines (Liu *et al*, 1996). These considerations indicate that transient JNK activation (or elevated basal JNK activity) may be important for mediating a survival response to stress, and that chronic JNK activation may contribute to an apoptotic response. A second aspect to consider is the crosstalk between different pathways. For example, the activation of AKT can suppress the apoptotic effects of activated JNK (Kim *et al*, 2002) or, alternatively, JNK may modulate other signalling pathways that mediate cell survival, such as NF-κB (Sanchez-Pérez *et al*, 2002). Target genes that have AP1 and NF-κB-responsive elements in their promoters could be differentially expressed according to the relative activity of the different pathways. Recently, Lamb *et al* (2003) reported that JNK activation, in response to TNF-*α*, increases together with c-jun and JunD levels. As a consequence, JunD induces c-IAP-2 transcription, while, at the same time, modulating apoptosis and cell survival in response to JNK activation.

NF-κB comprises a family of inducible transcription factors that act as regulators of the host immune and inflammatory responses. Also, they have been involved in protecting cells from apoptosis induced by chemotherapy agents, or cytokine treatment. NFKB is a heterodimer composed, among others, of p50 and p65/RelA subunits (Karin *et al*, 2002). In nonstimulated cells, NF-κB is found, mainly, in the cytoplasm, in association with a family of inhibitory molecules known as the IKBs. The activation mechanism of NF-κB involves phosphorylation of the IKBs via the IKB kinase (IKK) signalosome complex. Two different kinases that phosphorylate the IKKs have been described: NFKB-induced kinase and MEKK1. Once the IKBs have been phosphorylated, they are targeted for ubiquitin binding and subsequent degradation by the 26-S proteosome. Free p50/p65 heterodimers translocate to the nucleus, where they activate transcription of NFKB-responsive genes. Most of these genes such as BCLxl, XIAP, c-IAP-1,2 and FasL (Orlowski and Baldwin, 2002) are involved in apoptosis induction. Chemotherapy agents such as doxorubicin, cisplatin and taxol, among others, induce survival signals, depending on NFkB transcription and on the apoptosis pathways, driven mainly by JNK and p38.

A protein that is key in transducing the DNA damage signals to apoptosis is p53. Most chemotherapy agents, and *γ*-radiation, induce stabilisation and activation of p53. The transcription activity of p53 is important for its apoptotic functions, since it induces the expression of proteins directly involved in apoptosis, such as BAX, NOXA, PUMA, CD95, TRAIL-R1 and TRAIL-R2. Since p53 is mutated in nearly 50% of human tumours, induction of apoptosis by chemotherapy agents in these tumours can be compromised.

## STRATEGIES FOR OVERCOMING THERAPY RESISTANCE

Over the past decade, numerous strategies have been designed to modulate signalling pathways activated by oncogenes in an effort to inhibit cell growth in very specific ways. Since genetic alterations that accumulate in tumours favour survival of cells exposed to different stress conditions, the response to therapy frequently becomes severely compromised. New molecules that influence specific signalling pathways administered in combination with cytotoxic drugs have become the preferred strategies in preclinical and clinical studies in an attempt to overcome intrinsic drug resistance. We shall, now, proceed to describe some of these strategies.

Restoring p53 activity in tumour cells has a therapeutic potential because p53 is lost in many tumours and results in resistance to platinum compounds and radiotherapy. Different approaches have been tried in restoring wtp53 activity. These include gene transfer of wtp53 and chemical restoration of wt activity. Gene transfer has been accomplished by using replication-deficient adenoviral vectors that express wtp53. Different preclinical studies have demonstrated synergistic activity between re-expression of p53 and chemotherapy in the patient's response to therapy (Nielsen and Maneval, 1998). The combination of wtp53 gene transfer and chemotherapy with cisplatin, doxorubicin, 5-FU, methotrexate or etoposide in human xenograft tumour animal models of the lung, breast, head and neck, prostate and ovarian cancer produced significantly greater apoptosis and tumour growth suppression than any of the drugs alone (Lebedeva *et al*, 2003). These results have encouraged clinical studies in patients with advanced cancer. At the NIH database, there are, currently, 29 clinical trials employing wtp53 gene transfer in different types of cancers. Some of these trials show that NSCLC treated with cisplatin, vinorelbine or a combination of both resulted in stabilisation, and regression, of tumours. With respect to ovarian cancer, the one study that used cisplatin resulted in an increased median survival compared to chemotherapy alone. Although the results are promising, the limitation of this approach is linked to the development of new and improved vectors that are not toxic, nonimmunogenic and which can transfer the gene to a high proportion of cancer cells.

An alternative approach that has been used to restore p53 activity is the use of small molecules that modify the p53 mutant strain back to wildtype. CP-31398, a styrylquinazoline, restores a wild-type DNA-binding conformation of mutant p53, and is able to suppress tumour growth *in vitro* and *in vivo*. CP-31398 induces apoptosis in p53 mutant cancer cells by activating the bax/mitochodrial/caspase-9 pathway, suggesting that it can also sensitise tumour cells to cytotoxic drug treatment (Luu *et al*, 2002).

Targeting the growth factor receptors that are involved in the survival of cancer cells has become a potential strategy for apoptosis induction, either as a single treatment or in combination with traditional therapies. One potential therapeutic target is the epidermal growth factor receptor (EGFr) superfamily. Many different strategies that interrupt EGFr-dependent signalling have been investigated. Monoclonal antibodies have been developed that target members of the EGFr family. These include antibodies to EGFr and monoclonal antibodies to HER2. Cetuximab, a human-murine chimeric IgG monoclonal antibody, competitively binds to the extracellular domain of EGFr, thus preventing tyrosine kinase activation and inducing apoptosis (Herbst and Langer, 2002). Preclinical studies have demonstrated that, in cell lines expressing EGFr as well, there is an increase in the cytotoxic activity of chemotherapy and radiation (Robert *et al*, 2001). Clinical studies in patients with NSCLC and head and neck cancer are in progress using this antibody in combination with chemotherapy. Trastuzumab (Herceptin), a monoclonal antibody against the extracellular domain of HER2, was designed for use in breast cancers in which HER2 amplification occurs more frequently. A phase III clinical trial for patients with metastatic breast cancer with overexpression of HER2 demonstrated that the addition of trastuzumab to chemotherapy was associated with a delayed time to -progression of the disease (Azzoli *et al*, 2002). HER2 is found amplified in lung adenocarcinomas and large-cell carcinomas as well, and is predictive of poorer outcomes. Clinical trials with HER2-positive patients with advanced NSCLC failed to improve treatment outcome for most of the patients in the trial, although a small percentage, in whom HER2 was found to be positive by fluorescence *in situ* hybridisation (FISH), responded to treatment.

Tyrosine kinase inhibitors to EGF receptors have been developed that compete with and prevent the binding of adenosine triphosphate to the tyrosine kinase region. In preclinical studies, these agents cause inhibition of cell proliferation and induce apoptosis in EGFr-positive cell lines. Gefitinib (ZD1839, Iressa), one of the agents currently in the most advanced stage of development, has shown encouraging results in preclinical studies when combined with chemotherapy in NSCLC. The clinical trials in patients with stage III/IV NSCLC using gefinitib, in combination with gemcitabine and cisplatin, failed to demonstrate any improvement in survival rates. Although other clinical studies, with a more strict selection of patients, have been conducted, it is still not clear if the expression of EGFr is necessary for response, or whether its inhibition will represent a clinical benefit of treatment in NSCLC.

Therapeutic targeting of ras has been approached by inhibiting the covalent attachment of the farnesyl-isoprenoid group to the H-ras, K-ras and N-ras proteins, the first steps in the carboxy-terminal post-translational modification of these proteins. The farnesyltransferase inhibitors (FTIs) that have been developed to target RAS also inhibit the farnesylation of other proteins and some evidence indicates that the effects observed in tumour growth are due to inhibition of the RAS-related protein RHOB (Prendergast, 2001). This issue, however, remains unresolved at present. Farnesyltransferase inhibitors have shown important effects in growth and survival of some tumour cell lines *in vitro* and in xenografts in nude mice. Although the results of preclinical studies have been promising, clinical studies have faced several stumbling blocks in their efforts to demonstrate their usefulness in common cancers. Nevertheless, some interesting results have emerged from clinical trials in patients with chronic myeloid leukaemia and other haematological malignancies. Also, in preclinical studies, FTI-277 has been demonstrated to inhibit growth and induce apoptosis in drug-resistant myeloma cells as well as in gliomas when combined with radiotherapy. Interestingly, SCH66336 has been shown to enhance chemosensitivity of human and mouse melanoma cells to cisplatin, and to overcome STI571 resistance in BCR-ABL-positive cells. These results suggest that combination therapy may be more effective in patients with STI-571 resistance (Hoover *et al*, 2002).

The survival-signalling pathway, whose components are subjected to more frequent deregulation in cancer, is that of the PI3K/AKT pathway, and the consequences of this process contribute to malignancy progression as well as to treatment resistance. As such, chemical inhibitors of AKT have a potential use as suppressors of tumour growth. Early generation examples of this type of compound (wortmannin and LY294002 which inhibit the catalytic activity of p110) have been studied extensively in *in vitro* assays. LY294002 administration in mouse tumour models has been shown to confer antitumour activity and to enhance the cytotoxicity of treatment with taxol and radiotherapy (Hu *et al*, 2002). Interestingly, in human breast cancer cells, inhibition of PI3K by LY294002 blocks the resistance induced by ras transformation (Jin *et al*, 2003). The use of rapamycin, an immunosuppressant drug already being used in the clinic and which is an inhibitor of the AKT substrate, mTOR kinase, has opened up the possibility of indirectly inhibiting akt survival pathways. Derivatives of rapamycin, such as CCI-779, have been shown to inhibit the tumour growth of several PTEN-negative tumour cells *in vitro* and to increase the cytotoxic activity of traditional therapies, Neshat *et al* (2001). In view of the results obtained with wortmannin, LY294002 and rapamycin derivatives, several drug-discovery programs are underway. The search is for small-molecule inhibitors of PI3K and several other kinases in the pathway, including PDK1 and the integrin-linked kinase (ILK), which might be equally important in keeping the AKT pathway activated.

Activation of the NF-*κ*B-dependent transcription itself, or as a consequence of activated ras/PI3K/AKT pathway, occurs in several human tumours. Additionally, some antitumour agents such as paclitaxel and cisplatin, among others, have induced cell survival via this pathway. Adenovirus-mediated inhibition of NF-*κ*B elicited by the IKB super-repressor protein abrogate the chemoresistance of some types of tumours such as androgen-independent prostate cancer cells and glioma-derived cell lines (Orlowski and Baldwin, 2002). Work is in progress in several laboratories to isolate small molecules that inhibit the IKKs, and whose activities in some tumours lead to constitutive NF-*κ*B activation and in others, such as breast cancer, where IKK*α* plays an important role in proliferation. Finally, targeting of IAPs resulting in upregulation by means of the NFKB pathway has been approached via the activation of the apoptosis-ligand receptors of the Apo2/TRAIL, most normal cells being resistant to treatment with TRAIL while cancer cell lines are sensitive to Apo2/TRAIL. Chemotherapy and/or radiation could sensitise TRAIL-induced apoptosis *in vitro* as well as *in vivo*. More recently, smac/diablo-derived peptides, have been shown to sensitise tumour cells to Apo2/TRAIL or anticancer drug-induced apoptosis in gliomas *in vivo* (Fulda *et al*, 2002).

## FUTURE DIRECTIONS

**Figure 1:**
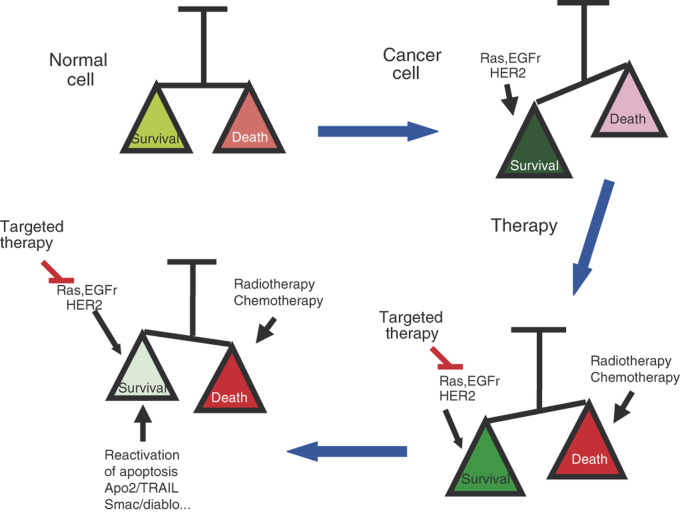
Combination therapy would shift the balance towards apoptosis in the response of drug-resistant tumours. Note: In normal cells, the balance among apoptosis and survival signals allow controlled homeostasis of the tissue. In cancer cells, survival signals triggered by oncogenic transformation favour uncontrolled growth. Traditional treatments such as chemotherapy and radiotherapy, together with targeted therapies, are still not sufficient to kill tumour cells efficiently due to the existence of antiapoptotic mechanisms in the tumour. Reactivation of the apoptotic machinery by strategies such as Apo2/TRAIl delivery would, probably, make the combination of traditional and targeted therapies more efficient.

At the preclinical as well as the clinical level, the problem of drug resistance is still unresolved. Many different approaches have been taken using tumour-derived cell lines and inhibiting signalling pathways activated by oncogenes that participate in cell survival by the administration of combinations of chemotherapy and radiotherapy (Figure 1

). One issue that remains unresolved is that of the relative importance of these pathways for tumour survival *in vivo*, and how they co-operate with genetic changes in the apoptotic machinery in order to ensure tumour progression. Hopefully, more knowledge of the genetic changes that contribute to malignancy in each tumour will be acquired soon, using genomic and proteomic techniques. This would also help in the identification of profiles of expression associated with drug sensitivity or resistance, and this information is used to decide the drug to be used as a first choice. With current knowledge, there are several candidate genes to be taken into consideration, whose activity would be predictive of poor response. Among them are AKT itself and genes that regulate AKT activity, such as PTEN. In this context, several additional aspects need to be taken in consideration. These involve the activity of genes implicated in DNA repair that can condition the efficiency of drug activity in target cells. Last, but not least, therapies that include inhibition of oncogene-dependent pathways in combination with traditional therapies and upregulation of apoptosis in tumours by alternative routes (Apo2/TRAIL, smac/diablo) will, undoubtedly, contribute to the reversal of resistant phenotypes.
